# Comparison of Capillary Electrophoresis and HPLC-Based Methods in the Monitoring of Moniliformin in Maize

**DOI:** 10.3390/foods14152623

**Published:** 2025-07-26

**Authors:** Sara Astolfi, Francesca Buiarelli, Francesca Debegnach, Barbara De Santis, Patrizia Di Filippo, Donatella Pomata, Carmela Riccardi, Giulia Simonetti

**Affiliations:** 1 Department of Chemistry, “Sapienza” University of Rome, P.le Aldo Moro, 5, 00185 Rome, Italy; sara.astolfi@uniroma1.it (S.A.); giulia.simonetti@uniroma1.it (G.S.); 2 Department of Food Safety, Nutrition and Veterinary Public Health, Italian National Institute of Health, Viale Regina Elena 299, 00161 Rome, Italy; francesca.debegnach@iss.it (F.D.); barbara.desantis@iss.it (B.D.S.); 3 Inail DIT-Via Roberto Ferruzzi, 38, 00143 Rome, Italy; p.difilippo@inail.it (P.D.F.); d.pomata@inail.it (D.P.); ca.riccardi@inail.it (C.R.)

**Keywords:** emerging mycotoxins, moniliformin (MON), food contamination, CE-DAD, HPLC-DAD/HPLC-MS-MS, risk assessment

## Abstract

Over the past few decades, scientific interest in mycotoxins—fungal metabolites that pose serious concern to food safety, crop health, and both human and animal health—has increased. While major mycotoxins such as aflatoxins, ochratoxins, deoxynivalenol, fumonisins, zearalenone, citrinin, patulin, and ergot alkaloids are well studied, emerging mycotoxins remain underexplored and insufficiently investigated. Among these, moniliformin (MON) is frequently detected in maize-based food and feed; however, the absence of regulatory limits and standardized detection methods limits effective monitoring and comprehensive risk assessment. The European Food Safety Authority highlights insufficient occurrence and toxicological data as challenges to regulatory development. This study compares three analytical methods—CE-DAD, HPLC-DAD, and HPLC-MS/MS—for moniliformin detection and quantification in maize, evaluating linear range, correlation coefficients, detection and quantification limits, accuracy, and precision. Results show that CE-DAD and HPLC-MS/MS provide reliable and comparable sensitivity and selectivity, while HPLC-DAD is less sensitive. Application to real samples enabled deterministic dietary exposure estimation based on consumption, supporting preliminary risk characterization. This research provides a critical comparison that supports the advancement of improved monitoring and risk assessment frameworks, representing a key step toward innovating the detection of under-monitored mycotoxins and laying the groundwork for future regulatory and preventive measures targeting MON.

## 1. Introduction

Mycotoxins are toxic secondary metabolites produced by various pathogenic fungal species, which commonly contaminate a wide range of food products worldwide [1]. In general, substrates rich in carbohydrates and lipids promote mycotoxin contamination. The foods most at risk include cereals, legumes, fresh and dried fruit, spices, cocoa, and green coffee. Globally, more than 25% of agricultural products are affected by mycotoxin contamination, although data often represent underestimations [2]. Maize (*Zea mays*) is one of the most widely cultivated cereal crops across the world, playing a crucial role in food and feed production. It is increasingly used in the infant food industry due to its nutritional profile and versatility [3], providing energy, carbohydrates, fiber, and some vitamins. Its hypoallergenic nature also makes maize a suitable and versatile option for infants with intolerance to other gluten-rich grains like wheat. Furthermore, maize is a staple food in many parts of the world, particularly in regions with limited access to other protein and nutrient sources. Its affordability, high energy content, and adaptability to different climates make it essential for food security in areas such as Latin America, parts of Asia, and sub-Saharan Africa [4,5]. In these regions, maize is widely used in traditional dishes such as porridges, flatbreads, and stews, playing a vital role in daily diets. However, despite its nutritional and cultural importance, heavy reliance on maize as a staple food can pose public health risks due to potential contaminants, such as mycotoxins, that may be present in improperly stored or processed grain.

In recent years, climate change has gradually altered the relationship between plant growth and related fungal diseases. The key factors influencing fungal life cycles, as well as their capacity to produce toxins, survive, and colonize crops, include temperature, water availability, moisture, and extreme conditions such as drought and desertification [6]. These factors have, on the one hand, contributed to the spread of new mycotoxins in different regions and crops. On the other hand, some fungi may not survive in extreme conditions, reducing the likelihood of crop infections [7]. Extensive research has focused on the effects of climate change on major mycotoxins, such as aflatoxins, deoxynivalenol, and fumonisins. Several reviews have documented how changes in temperature and moisture regimes influence the prevalence and distribution of these well-known toxins [8].

The presence of mycotoxins in food and feed has detrimental effects on human and animal health, posing a risk to a sustainable food supply [9,10]. Therefore, it is essential to conduct widespread monitoring [11] and establish preventive strategies aimed at inhibiting the production of toxigenic fungi and mycotoxin contamination during all stages of product management (cultivation, transport, storage, and processing and transformation) [12]. In addition, the control of contamination in agricultural products along the entire food chain is of fundamental importance. This applies not only to well-known mycotoxins (aflatoxins, ochratoxins, zearalenone, fumonisins, etc.), whose maximum levels have already been set for several foods and derived products [13,14], but also for the so-called “Emerging Mycotoxins” (EMs) [15]. Although not yet regulated, EMs have been identified and detected in food and feed representing a concern. Notably, EU Regulation 2023/915 [14] sets maximum levels for several regulated mycotoxins but does not cover emerging mycotoxins such as MON. This regulatory gap highlights the need for further data on MON occurrence and toxicity to support potential future legislation and ensure adequate consumer protection. Some of the most interesting EMs for the scientific community are produced by fungi from the *Fusarium (F)* genus, including MON. [16]. MON, produced by *F. proliferatum*, *F. subglutinans*, and *F. avenaceum*, is widely reported in Europe, America, and the Pacific region. MON was discovered by Cole [17] during a screening for toxigenic compounds produced by *F. verticillioides*, and it was subsequently isolated from maize seeds. This mycotoxin can accumulate in maize under specific environmental conditions during cultivation, transportation, storage, and processing. Accumulation is more likely when the crop is exposed to stress factors like drought or improper handling [18]. Recent regional studies, such as the multi-year investigation of MON occurrence in Serbian maize, have begun to shed light on the weather-dependent variability of MON contamination. These findings demonstrate that climatic factors, including temperature fluctuations and precipitation patterns, can significantly affect MON levels in crops. However, such studies remain limited in number and geographic scope, underscoring the need for more extensive monitoring and research across regions vulnerable to climate change [18].

MON is a mycotoxin that may pose significant health risks to both humans and animals. In its opinion, the European Food Safety Authority (EFSA) [19] was unable to establish an Acute Reference Dose (ARfD) for MON due to limitations in the available acute and subacute toxicity data. However, cardiotoxicity was identified as the critical effect for short-term exposure, and based on a rat study, a NOAEL of 6.0 mg/kg body weight was selected as a reference point.

**Figure 1:**
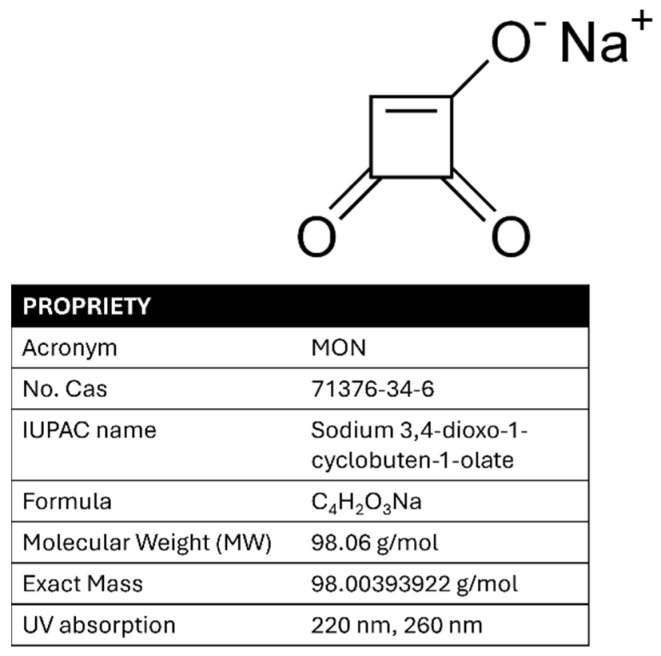
MON structure, name, acronym, properties, and UV spectrum.

For chronic exposure, the EFSA was unable to derive a Tolerable Daily Intake (TDI) due to a lack of sufficient long-term toxicity data. Nevertheless, haematotoxicity—observed as reductions in hematocrit and hemoglobin levels in a pig study—was identified as the key chronic effect. A Benchmark Dose Lower Confidence Limit for a 5% response (BMDL05) of 0.20 mg/kg bw/day was established as the reference point for chronic human exposure [19]. In addition, the lack of toxicokinetic data complicates both risk assessment and regulatory decision-making. Improved data collection, particularly on MON occurrence in cereals such as maize, is needed to support comprehensive human health risk assessments. Validated analytical methods for detecting MON are limited, highlighting the difficulty of implementing monitoring campaigns and official controls to ensure food safety. The analysis of MON differs significantly from that of other mycotoxins commonly found in maize flour as it is a small (MW: 98.06 g/mol), highly polar, and strongly acidic molecule (pKa approximately 1.7), making its determination particularly challenging. Unlike most of the well-analyzed mycotoxins (classical or emerging), which are soluble in most organic solvents, MON is highly soluble in water due to its polarity and exhibits limited solubility in other common polar solvents, such as acetonitrile and methanol. It is present in nature as sodium or potassium salt; however, water cannot be considered as an ideal extraction solvent as it often co-extracts numerous unwanted matrix components, even when ion-pairing reagents are used [20]. Therefore, a mixture of extraction solvents is generally preferred [21]. Its ultraviolet spectrum shows two characteristic absorption bands at approximately 260 nm and 230 nm, and it decomposes at 345–355 °C [22]. The structure, chemical name, acronym, physicochemical properties, and maximum UV absorption of MON are shown in Figure 1.

Given the complex chemical composition of real food matrices, it is always advisable to perform sample purification prior to analysis. Following extraction with polar solvents such as water, acetonitrile, and methanol in different proportions, depending on the nature of the sample, a purification step (commonly using Solid-Phase Extraction) is desirable to obtain a clean, suitable extract [23].

For instrumental analysis, liquid chromatography is the most appropriate technique for the determination of MON in food matrices. For its detection, Diode Array Detection (DAD) and mass spectrometry are commonly employed. Recently published works suggested the use of hydrophilic interaction liquid chromatography (HILIC) [23,24,25] for the analysis of small, polar molecules. Since MON is a charged molecule, zonal capillary electrophoresis (CZE) is also a suitable technique for its analysis. Capillary electrophoresis is a separation technique that offers the following advantages: (i) it is considered a green technique for the use of water microvolumes as a medium; (ii) it involves very low management effort and maintenance costs; and (iii) compared to other LC techniques, it requires shorter times to perform the whole analytical step. The peculiarity of this instrumental technique is that, depending on the chemical nature of the molecules, it offers the possibility of determining the analytes both by cathodic and anodic detection [26,27].

Studies focusing on MON are limited and current research remains inadequate. Very often, MON is reported to co-occur with other mycotoxins, such as deoxynivalenol zearalenone, fumonisins, beauvericine, and enniatins [28,29,30,31], potentially leading to synergistic toxicological effects.

To address existing knowledge gaps and enhance scientific understanding on diverse analytical approaches for detecting MON in maize and maize-based products, this study presents the development and validation of a multi-technique method for the determination and quantification of MON in maize flour samples. This work integrates three objectives of (i) optimizing and validating a robust analytical protocol by comparing three different techniques, (ii) conducting a preliminary monitoring survey to gather contamination data, and (iii) performing a targeted exposure assessment using the margin of exposure approach (MOE) to evaluate potential health risks associated with MON. By comparing multiple analytical techniques (capillary zone electrophoresis with DAD detection, HPLC with DAD detection, and HPLC coupled with tandem mass spectrometry), it was possible to improve detection accuracy for MON quantification in maize matrices. The method was subsequently applied to a limited set of samples to assess its practicality and reliability in real-world scenarios. The resulting contamination data enabled a preliminary estimation of dietary exposure, contributing to a better understanding of the potential risks linked to MON presence in maize-based foods.

By addressing methodological and data gaps, this study contributes to improving MON detection capabilities and supports evidence-based risk assessments for this emerging mycotoxin.

## 2. Materials and Methods

### 2.1. Chemicals and Reagents

Milli-Q water (Millipore Corporation, Burlington, MA, USA) was used. Organic solvents including acetonitrile (AcN) (Romil-UpSTM Ultra Purity Solvents, London, UK), methanol (CH_3_OH, MeOH), hexane, and formic acid (HCOOH) were obtained from Carlo Erba Reagents (France). Hydrochloric acid (37% HClaq) and ammonium formate (NH_4_HCO_2_) were also purchased from Carlo Erba Reagents, while sodium hydroxide (NaOH) was acquired from Agilent Technologies.

Background electrolytes (BGEs) included borax buffer (pH 9), phosphate buffer (pH 7), and phosphate buffer (pH 2.5), all supplied by Agilent Technologies (Santa Clara, CA, USA). The QuEChERS salt mixtures used for extraction (in 50 mL tubes) were composed of NaCl (1 g), MgSO_4_ (4 g), sodium citrate sesquihydrate (0.5 g), and sodium citrate (1 g), and were supplied by Waters (Waters S.p.A, Sesto San Giovanni, Milano, Italy). MON (1 mg) was purchased from Sigma-Aldrich (Milano, Italy) and stored at 4 °C until use.

The primary standard solution was prepared by dissolving 1 mg of MON in 2 mL of a 1:1 (*v*/*v*) mixture of acetonitrile and Milli-Q water. This stock solution was stored at −20 °C in the dark to prevent degradation. Working solutions were obtained by successive dilutions of the primary standard with acetonitrile–water (1:1, *v*/*v*) following filtration, and these were stored at 4 °C in amber tubes to minimize exposure to light and ensure stability.

### 2.2. Materials

Cartridges: For the best recovery, STRATA C18-E (55 μm, 70 Å, 100 mg/1 mL) and STRATA C8 (55 μm, 70 Å, 100 mg/1 mL) cartridges and C18 (55 um, 70 Å, 60 mg; Phenomenex^®^ Castel Maggiore Bologna, Italy) were compared.

The following three chromatographic columns were tested:

- Kinetex HILIC 50 mm × 2.1 mm 2.6 μm 100 Å (Phenomenex^®^ Castel Maggiore Bologna, Italy);
- Luna HILIC 150 mm × 2.0 mm 3 μm 200 Å (Phenomenex^®^ Castel Maggiore Bologna, Italy);
- Sequant ZIC^®^-p-HILIC 150 mm × 2.1 mm 5 μm (Merck, Sigma-Aldrich, Darmstadt, Germany).

The CE silica capillary was 50 cm L × 50 μm I.D. (Agilent Scientific Instruments, Santa Clara, CA, USA). The separation path between the injection zone and the detection window was 40 cm (LEF).

Filters of 0.45 µm (Millex HV, Millipore, MA, USA) and 0.22 µm (Millex^®^-CV 0.22 µm, Phenomenex^®^, Castel Maggiore Bologna, Italy) were used.

### 2.3. Instruments

#### 2.3.1. Capillary Electrophoresis System

The capillary electrophoresis (CE) system was integrated with a diode array detector (DAD; Agilent 7100, Agilent Scientific Instruments, Santa Clara, CA, USA). The sample injection was performed by an autosampler consisting of a mobile rack that can hold up to 50 vials. The pressure (50 mpsi) and time of injection (5 s) were set to achieve the best performance. The instrument was controlled by Agilent ChemStation 2010 software, which enables the setting of all operational parameters for the analysis and manages data processing.

#### 2.3.2. HPLC-UV

The HPLC-UV system featured a UV/VIS-DAD diode array detector with a variable wavelength range of 190–700 nm, using a deuterium and tungsten lamp (Nexera X3, SHIMADZU, Kyoto, Japan). The system included high-pressure pumps for solvent flow and an autosampler for automatic sample introduction. The dedicated software managed the separation methods, data acquisition, and results analysis, with various detector options available for specific applications.

#### 2.3.3. HPLC-MS/MS

A high-performance liquid chromatograph was used with a high-pressure mixer (Agilent 1260 Infinity II) coupled with a triple quadrupole mass spectrometer (API 2000 AB SCIEX; Applied Biosystems Foster City, CA, USA) equipped with an Electrospray Ionization (ESI) interface as the ionization source. Acquisitions were made in MRM mode after optimizing all the electrical parameters in the infusion (MON concentration = 10 µg/mL at 10 µL/min) based on Product Ion Scan experiments and selecting the significant precursor (PI) and fragment ions (FI).

### 2.4. Samples

To represent a scenario as realistic and diverse as possible, samples were randomly selected using the most common brands intended for the preparation of typical savory and sweet recipes for human consumption (Table 1). Ten samples of maize flour, all Italian in origin, were analyzed; these were made available by the Italian National Institute of Health (ISS) or, otherwise, purchased from supermarkets.

### 2.5. Sample Preparation

Before each analysis, all samples (1 kg) were subjected to homogenization using a blender. To obtain a homogeneous and representative sample, the analytical sampling was performed using the “quartering” method. This method involves flattening the sample from a cone shape, then dividing it into four equal parts. Two parts were discarded, while the remaining two were combined and aliquoted for further analysis. This procedure was repeated until a final 10 g sample was obtained.

**Figure 2:**
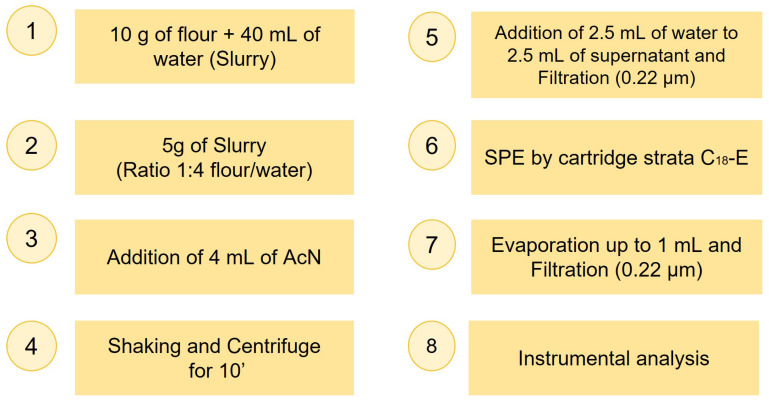
Scheme of the final procedure of the extraction and purification of MON from maize samples for a single aliquot.

Sample preparation for MON extraction and purification from maize involved optimizing some key steps, initially tested on standard or spiked solutions in a blank matrix. The procedure was then adapted to naturally contaminated maize samples. Due to the heterogeneous distribution of mycotoxins in flour, four to eight aliquots per sample were processed using the optimized method and then pooled and concentrated, and the final concentration was normalized by the number of aliquots. The optimization trials are described in the Results section. The final procedure adopted for a single aliquot is outlined in Figure 2.

The centrifuge used in this study (NF 400) was obtained from Nuve (Ankara, Turkey), and the shaker (Vortex—Genie 2) was purchased from Scientific Industries (Bohemia, NY, USA). The evaporator (Syncore Analyst) was obtained from Büchi Labortechnik (AG, Flawil, Switzerland).

### 2.6. Data Quality

Method validation followed Regulation [32,33] and Eurachem [34] guidelines. A set of three calibration curves was built to assess linearity (evaluated using R^2^ and further confirmed by back-calculating the concentrations of the calibration standards), apparent recovery, extraction recovery, and the matrix effect, as follows:

(1) curve in neat solvent;
(2) curve downstream (obtained spiking the extract of a blank sample);
(3) curve upstream (obtained spiking a blank sample before the extraction).

Each point of the curves was injected six times on the same day for repeatability (*RSD*_r_) and on three separate days for within-laboratory reproducibility (*RSD*_WR_) using the following formula:

$$RSD=\frac{standard\;deviation}{average\;value}\times \;100$$

The Limit of Detection (*LOD*) was calculated according to Wenzl [35], using the following formula:

$$xLOD=3.9\times \;\frac{s_{y,b}}{b}$$

where *S_y,b_* is the standard deviation of ten independent blank or pseudo blank signals, and *b* is the slope of the calibration curve at a concentration level near to the expected *LOD*. The Limit of Quantification (LOQ) was calculated as the lowest concentration level among the validated levels.

The apparent recovery (*R_A_*) was determined as the ratio between the angular coefficients of the two curves upstream versus neat solvent. This represents total recovery as it refers to the signal, influenced by both the matrix (sample composition) and the analytical procedure. The formula used for the calculation of *R_A_* [36] was as follows:

$$R_{A}=\frac{m_{upstream}}{m_{solvent}}$$

The matrix effect (Signal Suppression/Enhancement, *SSE*) is caused by interference from the matrix during the ionization phase of the target analytes. The presence of interfering substances in the matrix can result in either a decrease (suppression) or an increase (enhancement) in the recorded signal. *SSE* is expressed as the ratio of the angular coefficients of the curve prepared with the fortified extract (downstream) and that prepared in neat solvent, meaning it excludes the analytical process and exclusively evaluates the effect of the interferents on the signal of the target analytes. Thus:

$$SSE=\frac{m_{downstream}}{m_{solvent}}$$

Extraction recovery (*R_E_*) was adopted as a measure of the method’s efficiency that does not account for the effect of the matrix, calculated as follows [36]:

$$R_{E}\%=\frac{R_{A}}{SSE}\times 100$$

Selectivity was demonstrated by observing the eventual presence of interfering peaks at the MON retention or migration time ± 2.5% in ten blank samples and cecking significant interfering peaks of the mass transitions (in LC-MS/MS) of the analyte [37].

### 2.7. Risk Assessment

To interpret the results, a risk assessment was conducted using the Rapid Assessment of Contaminant Exposure (RACE) tool. This tool is based on the methodology proposed in the EFSA technical report “Risk evaluation of chemical contaminants in food in the context of RASFF notifications” [38]. The tool uses food consumption information from the EFSA Comprehensive European Food Consumption Database to provide estimates of acute and chronic exposure from single food items and compares the result with health-based guidance values or other relevant toxicological reference points.

The tool requires input parameters including the chemical substance, the food item (selected from a drop-down menu that uses the FoodEx2 classification system), the analytical value of the chemical substance, and the reference point for acute and/or chronic exposure. To provide clearly interpretable outputs, the tool reports the results for which there is no concern in green boxes and those for which the calculated value suggests a possible risk in red boxes; yellow boxes represent an intermediate scenario.

Both acute and chronic exposures were assessed. The food item selected was maize flour [Grains and grain-based products (L1), Cereal and cereal primary derivatives (L2), Cereal and cereal like flour (L3), Maize milled (L4), and Maize flour (L5)]. For the acute scenario calculations, a no-observed-adverse-effect level (NOAEL) of 6.0 mg/kg body weight was considered based on a subacute toxicity study. The calculated exposure outputs obtained from the RACE tool were subsequently divided into acute reference doses (ARfDs) and multiplied by 100 to obtain the overall ARfD percentage, with results greater than 100 indicating concern.

For chronic exposure, the results are expressed as a Margin of Exposure (MOE), which is a ratio of the dose at which a small but measurable adverse effect is first observed to the level of exposure to the substance considered. A small MOE represents a higher risk than a larger MOE. The results were classified as no risk (low probability of adverse health effects or low concern for public health), potential risk, or risk. To assess the potential chronic risk from MON exposure, the MOE was calculated as the ratio of the lowest BMDL_05_ value of 0.20 mg MON/kg body weight per day based on hematological adverse effects and chronic dietary exposure estimates for humans [19].

## 3. Results

The optimization of the method included both the instrumental conditions (evaluated using a standard solution) and the sample preparation process (assessed using spiked samples), applying all three techniques in both cases.

### 3.1. Optimization of CE Operational Conditions

To optimize the operational conditions for the analysis, various parameters influencing electrophoretic separation were adjusted. These parameters included the applied voltage, injection conditions, and the nature and concentration of the buffer.

#### 3.1.1. Voltage and Buffer

The applied voltage affects both the current intensity in the capillary and the analysis time. Initially, positive potentials were tested in cathodic mode, ranging from +18 kV to +32 kV using a basic buffer of 50 mM sodium tetraborate (Borax) at pH 9.3 to enhance the electroosmotic flow in the electrophoretic migration. The difficulty in the migration of the analyte led to the use of a less basic buffer, specifically sodium phosphate 50 mM at pH 7.0. Despite varying the applied potential, the results were still unsatisfactory. The best results were achieved using an acidic buffer (50 mM sodium phosphate at pH 2.5) and a negative potential, which allowed for optimal migration of MON, driven solely by electrophoretic flow, thereby eliminating the influence of electroosmotic flow. The instrumentation used was equipped with a UV-DAD spectrophotometer. Two optimal wavelengths were selected, 220 nm and 260 nm, corresponding to the maximum absorption peaks of MON (see Figure 1).

#### 3.1.2. CE Injection Conditions

The injection parameters were optimized for electrophoretic analysis. Increasing pressure and time results in more intense peaks but excessive pressure can cause leakage and deposition on the electrodes, while prolonged injection times may cause capillary clogging. Optimal conditions were found at 50 mpsi for 5 s. Using a non-functionalized fused silica capillary and an alkaline BGE-forming cathodic electroosmotic flow, sample injection was performed at the anode. This anodic detection combined with cathodic injection provided the best results. The final optimized instrumental parameters are reported in Table 2.

### 3.2. Chromatographic Column Optimization

Three columns were tested to optimize the HPLC separation—Kinetex HILIC, LUNA HILIC, and ZIC-p-HILIC. Initial tests with the Kinetex HILIC column using 60 mM ammonium formate (phase A) and acetonitrile as the mobile phases (phase B) showed a MON peak eluting before the dead volume. Varying the flow rate (0.3, 0.2, and 0.1 mL/min) and the mobile phase compositions (A: 5% and B: 95%, and A: 2% and B: 98%) did not improve the separation. Tests with the second column, LUNA HILIC, using different standard concentrations and elution conditions were unsatisfactory due to peak splitting, despite good retention. Finally, the ZIC-p-HILIC column proved to be the best option. Chromatographic runs with this column were performed isocratically at different mobile phase ratios (A: 5% and B: 95%, A: 10% and B: 90%, and A: 15% and B: 85%, were A = 60 mM ammonium formate and B = acetonitrile) at 25 °C and at different flow rates (0.3, 0.2, and 0.1 mL/min). The optimal conditions were achieved with a flow rate of 0.15 mL/min with 10% phase A and 90% phase B, which enhanced both sensitivity and peak shape. The autosampler temperature was set at 25 °C and 5 μL was injected. The chromatographic peak was well defined, no longer split, and eluted with a retention time of 7.5 min. The final optimized instrumental parameters are reported in Table 3.

### 3.3. Optimization of the Mass Spectrometry Parameters

Table 4 shows all the optimized mass spectrometric parameters. The acquisitions were performed in negative ionization mode with an Ion Spray potential (IS) of −5000 V, and the nebulizer, curtain gas (CUR), and collision gas (CAD) were set to 30, 20, and 4 psi, respectively. The source temperature was set to 350 °C.

### 3.4. Optimization of Sample Preparation

Sample preparation involved analyzing both upstream and downstream spiked samples in triplicate at different concentrations (see Table S1). The most critical steps were (1) extraction, (2) purification, and (3) evaporation.

STEP 1: Extraction

The objective was both to achieve good homogenization of the flour sample to ensure representative analysis, given the uneven distribution of mycotoxins, and to increase the efficiency of MON extraction from the flour. With this aim, various ratios of the matrix with water were tested including 1:1, 2:3, and 3:5, but the best result was achieved with a 1:4 ratio (10 g of flour to 40 mL of water). This ratio improved the extraction of MON, making the protocol simpler and more sustainable.

Next, 5 g of this slurry were treated with 4 mL of acetonitrile (1:1 with slurry water). The sample was shaken for 10 min, centrifuged twice, and the supernatant concentrated using a rotavapor. The direct analysis of the supernatant, after concentration, revealed significant background noise, likely due to matrix interference compounds. Traditional protocols, such as QuEChERS liquid–liquid extraction conventionally used for mycotoxin analysis [39], did not improve MON recovery (around 10%). This low efficiency is primarily due to MON’s high solubility in water, leading to its substantial loss during the extraction process.

STEP 2: Purification

To improve detectability, a purification approach was followed by testing three different SPE cartridges in filtration mode, preparing one upstream (MON spiked before SPE) and one downstream (MON spiked after SPE) sample, as follows:

(1) Strata C8;
(2) C18;
(3) Strata C18-E.

Cartridges were activated with 1 mL of acetonitrile and washed with 1 mL of deionized water. The Strata C18-E cartridge showed the best recovery rates (see Supplementary Material Table S2). The other two cartridges were discarded since they showed a recovery of less than 50%.

STEP 3: Evaporation

Two sample evaporation methods were tested. In Method 1, the sample evaporated to dryness, resulting in a loss of up to 60% of the MON. In Method 2, the sample volume was reduced to 1 mL, leading to a loss of only 10%. Due to its significantly lower loss, Method 2 was selected for the final procedure.

### 3.5. Recovery

The total recovery estimate was performed by adding 150 µL of MON (100 µg/mL) to 10 g of blank maize flour (upstream). The aliquots were left to rest overnight. The following morning, each aliquot was subjected to the procedure shown in Figure 2 and the results compared with a post-purification spiked sample (downstream). The recovery results are shown in the first column of Table S2. The optimized protocol minimized sample loss, with recoveries > 75%, confirming the excellent performance of the proposed procedure.

### 3.6. Data Quality

The detailed results of the method performance and data quality analyses are provided in the Supplementary Material. Table S1 shows the concentration range of each curve and the R^2^ values, which were always ≥ 0.995, demonstrated good linearity. Table 5 shows the *LOD*, LOQ, intra- and inter-day repeatability, and matrix effects of the three methods.

As expected, the limits of detection (*LOD*s) obtained using CE-DAD were higher than those achieved using HPLC-MS/MS in MRM mode. Nevertheless, both the *LOD*s and limits of quantification (LOQs) obtained using these techniques were lower or comparable to those reported in other studies [24,26,40,41]. Conversely, the poorest *LOD* obtained using HPLC-UV did not allow for the detection or quantification of MON in the real samples.

Repeatability and within-laboratory reproducibility were overall excellent, with values below 8.0% and 12%, respectively. Table S2 shows recovery experiments simulating real sample conditions. These were conducted by spiking blank maize flour samples with MON (both upstream and downstream, see Section 3.4) and comparing the results with calculated recovery efficiency (RE) and apparent recovery (RA) values. The recovery values in the first column align well with the RE, while discrepancies with the RA are attributed to matrix effects; a positive matrix effect (signal enhancement) was observed both for HPLC-UV and HPLC-MS/MS, whereas a negative matrix effect (signal suppression) was noted for CE-DAD.

All blank samples analyzed using CE-DAD and HPLC-MS/MS showed no interfering peaks at the retention or migration time of MON, confirming the high selectivity of these methods in contrast to HPLC-UV reported in Figure S1b.

## 4. Discussion

### 4.1. CE vs. HPLC

The real samples, listed in Table 1, subjected to the procedure shown in Figure 2, were analyzed to detect the possible presence of MON. Although HPLC-UV initially showed promising results with standards and with spiked samples at 1–15 μg/g, it proved not suitable for the analysis of real samples. Due to interference and high background noise between 7 and 10 min, the HPLC-UV technique was unsuitable in most cases (see Supplementary Material, Figure S1). Therefore, results are reported only for th HPLC-MS/MS and CE-DAD analyses.

**Figure 3:**
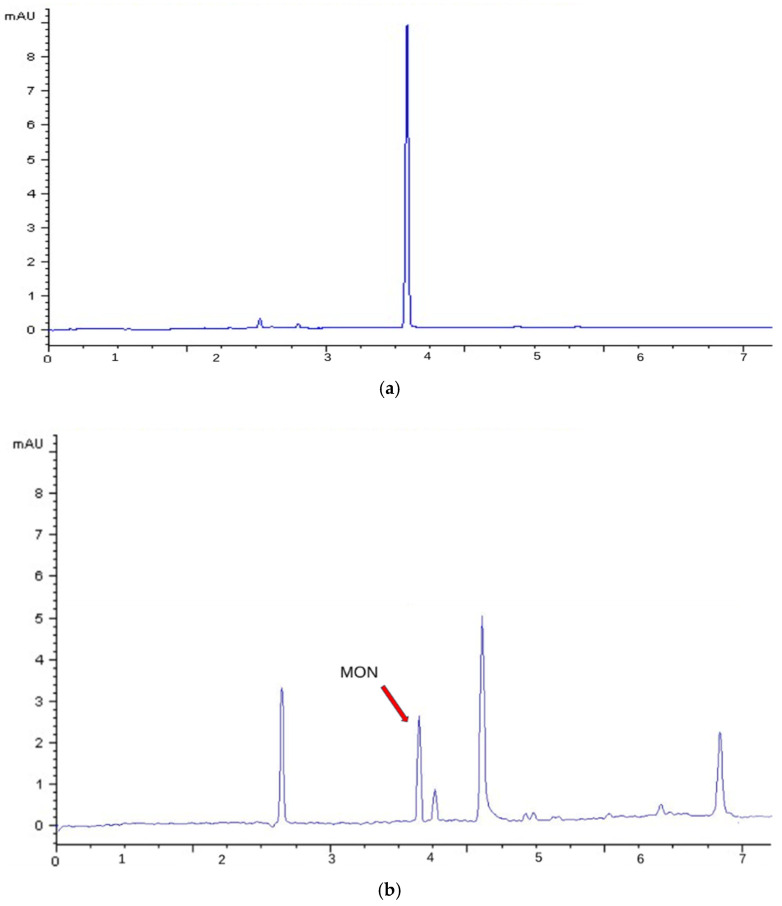
CE-DAD (260 nm) electropherogram obtained with the optimized conditions reported in Table 2: (**a**) Standard solution of MON at a concentration of 1 μg/mL (migration time = 3.9 min); (**b**) Naturally contaminated sample E1.

Figure 3 shows the electropherogram of (a) a MON standard (1 μg/mL) and (b) a naturally contaminated sample (E1) obtained using CE-DAD. By acquiring in anodic mode, at pH 2.3, migration is purely electrophoretic and the contribution from electroosmotic flow is eliminated. This acquisition enhanced selectivity, allowing for the detection of only anionic species. Under this condition, MON exhibited a migration time of 3.9 min.

**Figure 4:**
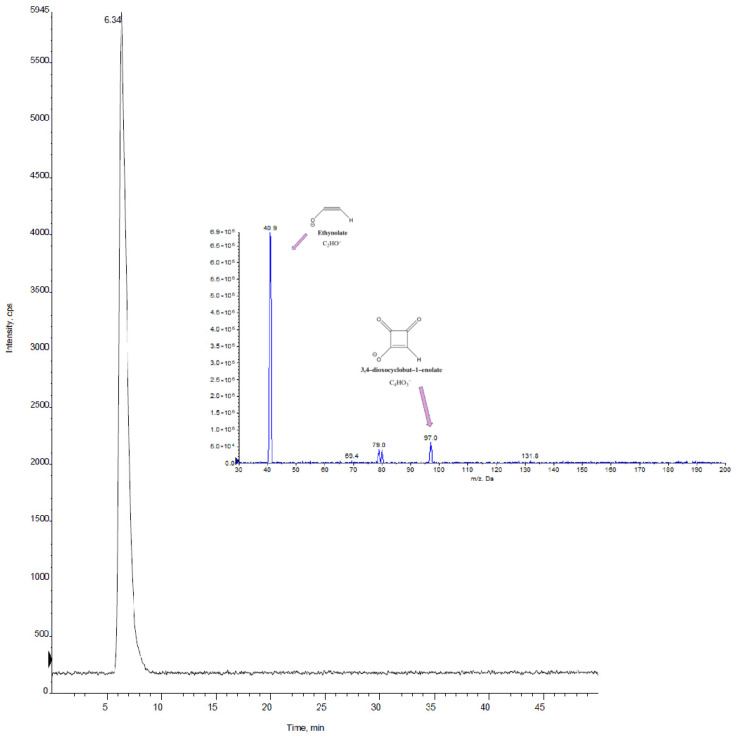
HPLC-MS/MS chromatogram in MRM mode for sample C2 and MS/MS spectrum of MON obtained with the optimized conditions reported in Table 4.

Figure 4 displays the chromatogram of a real sample (C2) obtained using HPLC-MS/MS (retention time = 6.3 min), and the MS/MS spectra of MON are reported.

For the identification of the analytes of interest using LC-MS/MS, the criteria outlined in the DG SANTE guidelines on the identification of mycotoxins and plant toxins in food and feed [37] were followed. The requirements for the separation were met, with a retention time ± 0.1 min compared to the average time obtained for the injections of standards in pure solvent, and for the mass spectrometry, which requires monitoring characteristic transitions with an ion ratio deviation of no more than ± 30% compared to the average value calculated for the standards injected in pure solvent.

In general, the advantages of CE over HPLC are its high resolution, low sample volume, fastness, efficiency, versatility, cost-effectiveness and, above all, eco-friendliness. In this specific case, CE-DAD was more efficient compared to HPLC-DAD due to the narrower peak width. On the other hand, mass spectrometry compared to UV detection enables sensitivity, molecular identification, reduced sample complexity, enhanced quantification, and structural insights. The best compromise could be capillary electrophoresis–mass spectrometry (CE–MS), which combines powerful separation with a sensitive identification, making it highly effective for complex matrices.

### 4.2. Application for Real Maize Samples

As previously noted, mycotoxins tend to develop unevenly within feed and flour, following a heterogeneous distribution pattern. Therefore, despite the preliminary homogenization of the sample and quartering, it is difficult to quantitatively determine the true contamination level in food matrices. To address this issue and obtain reliable results, four to eight aliquots were prepared for each sample. All aliquots underwent the optimized procedure, were pooled together, and then concentrated by evaporation. The values obtained from the instrumental analysis were subsequently normalized by division by the number of combined aliquots. Table 6 shows the comparison of the MON concentrations in the analyzed flours measured using CE-DAD and HPLC-MS/MS. All samples were processed in triplicate.

Based on the results presented in Table 6, mass spectrometry enabled the determination and quantification of MON in 7 out of 10 samples, with a content ranging between 0.06 to 0.4 μg/g. Even the sample labelled as organic agriculture (C5) displayed a MON concentration value consistent with samples C1, C4, and C7. Capillary electrophoresis in anodic mode demonstrated strong potential for rapid screening at concentrations equal to or greater than 0.1 μg/g. 

The developed analytical methods proved to be robust, as both capillary electrophoresis coupled to DAD and liquid chromatography coupled with mass spectrometry provided consistent results for the MON content in samples C1, C2, C3, and E1. However, due to the higher limits of detection of CE-DAD, it was not possible to quantify the mycotoxin levels in the other samples analyzed.

Precooked maize flour (samples C6 and F2) had lower levels of MON compared to raw maize. This could be due to processing treatments, such as heat or cooking at high temperatures, that could reduce any contaminant present in the raw product. However, effectiveness depends on the type of mycotoxin and the specific processing conditions [42,43,44,45]. This observed reduction may have implications for actual dietary exposure to MON, which could be overestimated if only raw flour concentrations are considered.

Given the limited occurrence data for MON in polenta flour and the lack of health-based guidance values, the findings of this study highlight the importance of implementing proper agricultural, storage, and processing methods to minimize contamination risk. In previous research on MON in maize, most studies have focused on different parts of the plant. Sørensen [25] examined entire maize plants derived from North Europe, reporting concentrations between 1 and 12 ng/g. Scarpino [46] focused on maize grains from Northwest Italy over a four-year period (2008–2011), reporting higher MON concentrations ranging from 4 to 2606 ng/g. This comparison shows how the type of sample analyzed can significantly influence the detected MON levels. In comparison, few studies have focused on MON contamination in flour. The results obtained for the maize flours analyzed in this study are comparable to those reported by Bertuzzi [47] and Herrera [21], confirming that the MON levels detected in processed maize products fall within a similar range, regardless of geographical origin. In particular, Bertuzzi [47] reported concentrations ranging from 92 to 343 μg/kg in maize flour samples from Italy, while Herrera [21] found levels of 171 and 201 μg/kg in two polenta samples from the Netherlands and Germany, respectively. In a recent paper, comparable ranges were also found in flour from sub-Saharan Africa (Namibia), where the occurrence of MON ranged from 3.3 to 723 μg/kg across 48 samples of flour consumed by children from rural households in Oshana [48].

### 4.3. Risk Assessment

A deterministic dietary exposure assessment was conducted using the RACE tool provided by the EFSA. The estimations were carried out to evaluate acute and chronic exposure scenarios to selected contamination values of MON. To run the RACE tool, maize flour mean consumption values were extracted from the EFSA Comprehensive European Food Consumption Database in Exposure Assessment [49]. Calculations were made for the analytical results of 0.4 μg/g obtained for samples C2 and C3, and, with the aim of evaluating possible critical scenarios, the calculations were also made considering a MON concentration of 1.5 μg/g. The RACE tool results for both acute and chronic exposure are calculated for the total population and consumers only for different age groups in Europe. Table 7 shows the acute output values for consumers only. All results, expressed as a percentage of the ARfD, are quite far from the critical value of 100, thus indicating a safe scenario.

Table 8 shows the chronic output for consumers only, with the results expressed as MOE. The MOE approach highlights a concern when calculated values for the population groups are below 100, representing the reference for substances without carcinogenic effects, as in the case of MON. Values in the range of 100 to 1000 represent an intermediate risk scenario, while no concern is underscored when MOE values are above 1000. The results reported in Table 8 indicate a general low risk to human health for both contamination levels except for infants and toddlers at a contamination level of 1.5 μg/g, which yielded MOE values below 100. For these age groups, the difference in body weight is a factor strongly influencing exposure, given that the contamination level used for calculation is the same for all age groups, and children have a consumption rate that is generally higher with respect to the body weight when compared to adult population groups.

As previously mentioned, thermal processing can significantly reduce MON levels. In addition, MON degrades under conditions like those found in traditional nixtamalization (an alkaline cooking process used in maize preparation), underscoring its instability during such processes [43,44]. Given these findings, the risk characterization scenarios outlined above may represent an overestimation of human exposure, particularly considering that maize flour is typically subjected to additional processing steps, such as polenta preparation, pasta production, or baking, prior to consumption. These processes are likely to further reduce MON levels, thereby lowering actual dietary intake compared to estimates based on raw or unprocessed flour.

## 5. Conclusions

This study addresses the growing interest in identifying and analyzing emerging mycotoxins in cereal-based food products, with a specific focus on MON—a significant contaminant frequently detected in maize. Due to its distinct chemical properties, MON poses considerable analytical challenges. The increasing adoption of baby-led weaning (BLW) together with the widespread inclusion of maize in infant diets raises concerns about early-life exposure to mycotoxins, particularly in areas where inadequate farming and storage practices may increase the risk of contamination. To better assess and manage this risk, a sample preparation protocol was developed and optimized to reduce matrix complexity and improve analytical reliability. Three analytical methods (CE-DAD, HPLC-UDAD, and HPLC-MS/MS) were evaluated and compared. 

Extraction recoveries ranged from 70% to 80%. Capillary electrophoresis in anodic mode demonstrated strong potential for rapid screening at concentrations equal to or greater than 0.1 μg/g, owing to its low background noise and consistent qualitative and quantitative performance. Conversely, HPLC-UV was limited by complex chromatograms and was ultimately deemed unsuitable. HPLC-MS/MS proved to be the most effective method, being suitable and optimal for confirmation methods and offering high sensitivity and precision for the trace-level detection of MON. 

Comparing multiple techniques not only improved detection accuracy but also enabled the cross-validation of results. The analysis of the maize samples supported methodological comparison and gave a general idea of contamination levels in the maize flour, helping to calculate exposure scenarios and identify critical contamination thresholds. Notably, the versatility of the proposed method, rooted in its mixed extraction solvent and SPE workflow that retains structurally diverse mycotoxins, offers a basis for future multi-toxin screening strategies. With minor adjustments, this approach could be expanded to include other polar mycotoxins. This conclusion is based on previous studies in which the use of a water–acetonitrile mixture, as employed in the extraction step in this study, is a widely accepted and effective as a solvent system for extracting various mycotoxins, including other polar compounds.

The dietary risk assessment carried out with the EFSA RACE tool indicates a general low risk to human health for both contamination levels, except for a chronic scenario for infants and toddlers at a contamination level of 1.5 μg/g, which yielded MOE values below 100. These findings highlight the need to advance food safety practices, refine analytical monitoring tools, and implement comprehensive regulatory measures to effectively manage the risks associated with both emerging and unregulated mycotoxins.

## Figures and Tables

**Table 1:** Maize flour samples analyzed.

Sample Code	Description
C1	Pure maize grown, dried, and finely ground (supplied by the ISS).
C2	Yellow maize flour produced through the milling of domestic maize. Used both for food intended for human consumption and for feed intended for animals (supplied by the ISS).
C3	Classical maize flour obtained from sustainable farming practices, respecting the environment and local regulations (supplied by the ISS).
C4	Maize guaranteed to have a maximum aflatoxin content of 4 ppb of and a maximum deoxynivalenol content of 1 ppm (supplied by the ISS).
C5	Flour made from pure maize from organic farming, dried and ground with a fine grain size.
C6	Precooked maize flour produced for making traditional polenta. This flour is made by cooking, drying, and grinding maize kernels. The process reduces cooking time as it only needs to be boiled briefly.
C7	“Bramata” yellow maize flour characterized by a coarser grind compared to fine maize flour, making it ideal for traditional polenta preparation.
E1	Maize flour of high-quality, traditionally milled stone-ground, made from locally sourced Italian maize and free from chemical additives. Ideal for making polenta and other recipes.
F1	Whole meal maize flour ideal for making polenta, bread, or other gluten-free recipes.
F2	Precooked (steam-cooked) 100% Italian maize flour. A quick and easy-to-prepare maize flour product for making polenta.

**Table 2:** Optimized conditions for capillary electrophoresis.

Potential	−18 kV
Current	32 μA
Buffer	Phosphate 50 mM pH = 2.5
Capillary	Silica bare ID 50 μm × 50 cm L
λ of detection	220/260 nm LEF 40 cm
Injection condition	Pressure 50 mpsi for 5 s

**Table 3:** Final instrumental parameters optimized for HPLC-UV.

Mobile Phase	Ammonium Formate 60 mM (10%) e Acetonitrile (90%)
Flow speed	0.15 mL/min
Temperature	25 °C
Injection volume	5 μL
Time of the analysis	10 min
Column	ZIC-p-HILIC 5 μm 150 × 2.1 mm
DAD detection	220,260 nm

**Table 4:** Optimized conditions for Precursor (PI) and Fragment ions (FI) of moniliformin (MW molecular weight 98 g/mol) and electrical parameters of the mass spectrometer.

Compound	PI (*m*/*z*)	FI (*m*/*z*)	EP (V)	FP (V)	CE (eV)	CXP (V)	DP (V)
MON	97 (M-H)^-^	41	−10	−200	−25	−5	−10
MON	97 (M-H)^-^	97	−10	−200	−25	−5	−10

DP, declustering potential; EP, entrance potential; FP, focusing potential; CE, collision energy; CXP, collision cell exit potential.

**Table 5:** *LOD*, LOQ, intra- and inter-day repeatability, and matrix effects of the three methods.

	*LOD* (μg/g)	LOQ (μg/g)	Intra-Day Repeatability	Inter-Day Repeatability	Matrix Effect
HPLC-DAD (220 nm)	0.4	1.3	5.2	7.9	1.6
HPLC-DAD (260 nm)	0.4	1.3	3.5	5.2	
CE-DAD (220 nm)	0.1	0.4	5.1	9.7	0.8
CE-DAD 260 nm)	0.1	0.4	8.0	12.0	
HPLC-MS/MS	0.003	0.01	4.0	6.2	1.8

**Table 6:** Comparison of moniliformin concentrations in analyzed flour samples obtained using CE-DAD and HPLC-MS/MS methods.

Sample	HPLC-MS/MS (μg/g ± SD)	CE-DAD (μg/g ± SD)
C1	0.09 ± 0.0018	0.10 ± 0.0050
C2	0.4 ± 0.0120	0.4 ± 0.0480
C3	0.4 ± 0.0160	0.4 ± 0.0240
C4	0.06 ± 0.0024	<*LOD*
C5	0.08 ± 0.0024	<*LOD*
C6	<*LOD*	<*LOD*
C7	0.06 ± 0.0024	<*LOD*
E1	0.10 ± 0.0050	0.10 ± 0.0090
F1	<*LOD*	<*LOD*
F2	<*LOD*	<*LOD*
		data

**Table 7:** Comparison of exposure to the toxicological reference point showing the highest output value expressed as acute % output for consumers only.

Population Group (Year)	Output Mean, % (MON 0.4 μg/g)	Output Mean, % (MON 1.5 μg/g)
Infants (0–1)	0.022	0.081
Toddlers (1–3)	0.026	0.096
Other children (3–10)	0.018	0.066
Adolescents (10–18)	0.008	0.032
Adults (18–65)	0.007	0.025
Elderly (65–75)	0.005	0.020
Very elderly (>75)	0.007	0.026

**Table 8:** Comparison of exposure to the toxicological reference point showing the calculated exposure value and the lowest output value expressed as MOE.

Population Group (Year)	MOE Mean (MON 0.4 μg/g)	MOE Mean (MON 1.5 μg/g)
Infants (0–1)	310	83
Toddlers (1–3)	261	70
Other children (3–10)	378	101
Adolescents (10–18)	664	177
Adults (18–65)	989	264
Elderly (65–75)	1244	332
Very elderly (>75)	938	250

## Data Availability

Data will be made available on request.

## Supplementary Materials

The following supporting information can be downloaded at: https://www.mdpi.com/article/10.3390/foods14152623/s1, Table S1: R^2^ and concentration range of each curve; Table S2: Recoveries (first column) obtained using HPLC-UV, CE-DAD, and HPLC-MS/MS by adding MON to blank maize flour upstream and downstream at a concentration of 10 μg/g (*RSD* < 10%) compared to RE and RA; Figure S1: HPLC-UV (260 nm), obtained with the optimized conditions reported in Table S1 (a) Standard solution of MON at a concentration of 5 μg/mL (retention time = 7.5 min) (b) Naturally contaminated sample (E1).
