# Supplementary material for: Comparison of Capillary Electrophoresis and HPLC-Based Methods in the Monitoring of Moniliformin in Maize
Source: Foods. 2025 Jul 26;14(15):2623. doi: 10.3390/foods14152623 (PMC12345993; doi:10.3390/foods14152623)
Supplement: Supplementary file 1 [file foods-14-02623-s001.zip › foods-3765305-supplementary.pdf]

SUPPLEMENTARY MATERIAL

Monitoring Moniliformin in Maize: A Comparative Analytical Study of Capillary Electrophoresis versus HPLC-Based Methods

Sara Astolfi[1], Francesca Buiarelli*[1], Francesca Debegnach[2], Barbara De Santis[2], Patrizia Di Filippo[3], Donatella Pomata[3], Carmela Riccardi[3], Giulia Simonetti[1]

1 Department of Chemistry, "Sapienza" University of Rome, P.le Aldo Moro, 5, Rome, 00185, Italy
2 Department of Food Safety, Nutrition and Veterinary Public Health, Italian National Institute of Health, Viale Regina Elena 299, 00161 Rome, Italy

3 Inail DIT-Via Roberto Ferruzzi, 38, Rome, 00143, Italy

Table S1. R^2 and concentration range of each curve.

	Solvent curve		Matrix matched calibration curve upstream		Matrix matched calibration curve downstream	
	R^2	Range (µg/mL)	R^2	Range (µg/g)	R^2	Range (µg/g)
HPLC-DAD (220nm)	0.998	0.6-15	0.996	1.3-15	0.995	1-15
HPLC-DAD (260nm)	0.997	0.6-15	0.994	1.3-15	0.997	1.3-15
CE-DAD (220nm)	1	0.2-10	0.998	0.4-10	0.995	0.2-15
CE-DAD (260nm)	0.999	0.2-10	0.997	0.4-10	0.997	0.2-15
HPLC-MS/MS	0.997	0.06-1.2	0.991	0.01-1.3	0.997	0.008-1.3

Table S2. Recoveries (first column) obtained in HPLC-UV, CE-DAD and HPLC-MS/MS by adding Moniliformin to blank maize flour upstream and downstream at10µg/g (RSD<10%) compared to RE and RA.

	Recovery %	R_E %	R_A %
HPLC-DAD (220nm)	70	74	121
HPLC-DAD (260nm)	80	78	125
CE-DAD (220nm)	77	81	66
CE-DAD (260nm)	79	81	64
HPLC-MS/MS	72	80	143

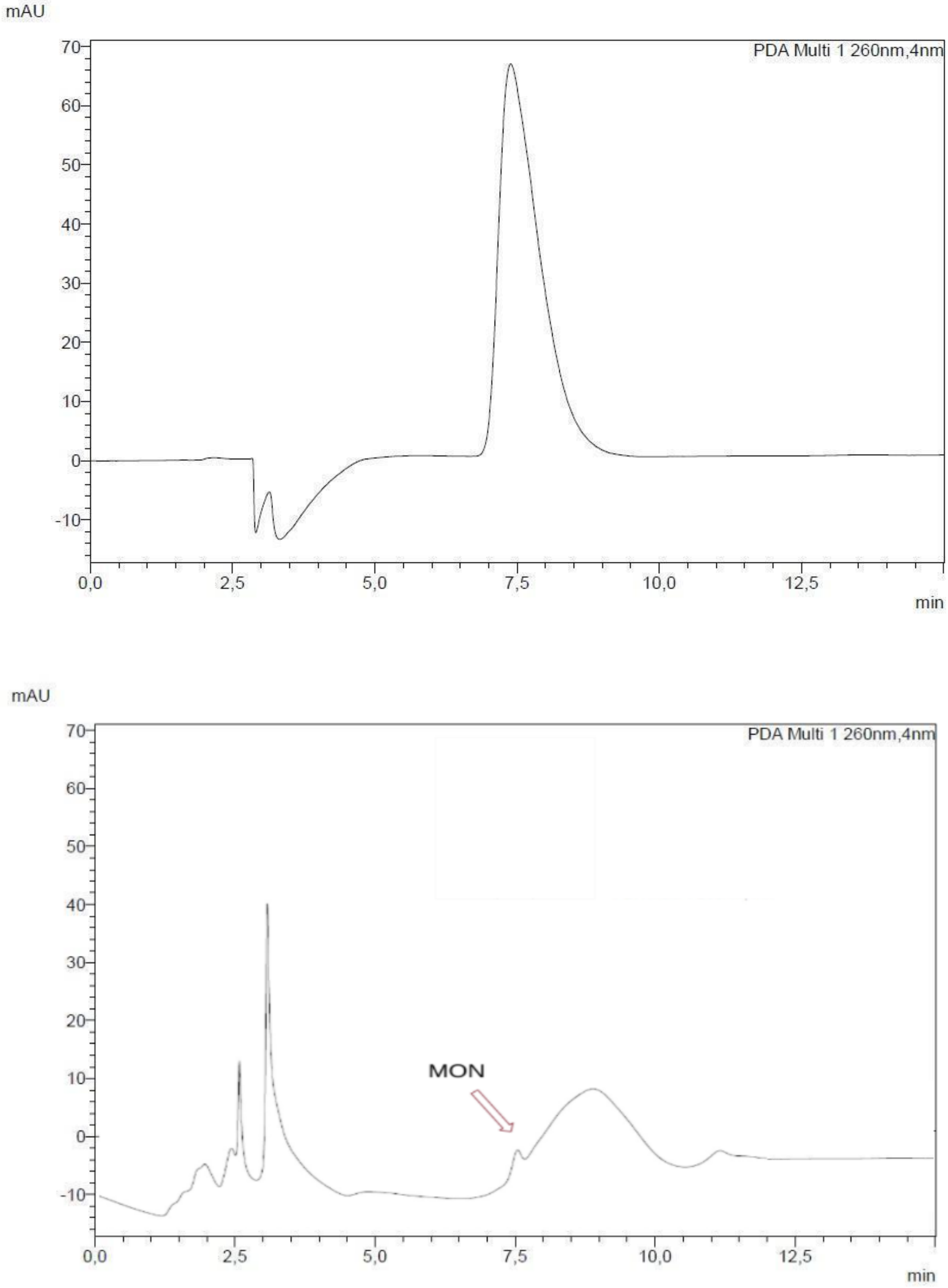

b)

Figure S1. HPLC-UV (260 nm), obtained with the optimized conditions reported in Table S1 a) Standard solution of Moniliformin at a concentration of 5 μg/mL (retention time 7.5minutes) b) Naturally contaminated sample (E1)
